# *In silico* investigation of the short QT syndrome, using human ventricle models incorporating electromechanical coupling

**DOI:** 10.3389/fphys.2013.00166

**Published:** 2013-07-05

**Authors:** Ismail Adeniran, Jules C. Hancox, Henggui Zhang

**Affiliations:** ^1^Computational Biology, Biological Physics Group, School of Physics and Astronomy, The University of ManchesterManchester, UK; ^2^School of Physiology and Pharmacology, and Cardiovascular Research Laboratories, School of Medical Sciences, University of BristolBristol, UK; ^3^School of Computer Science and Technology, Harbin Institute of TechnologyHarbin, China

**Keywords:** short QT syndrome, stretch-activated channel, mechanical contraction, 3D model, human ventricles

## Abstract

**Introduction:** Genetic forms of the Short QT Syndrome (SQTS) arise due to cardiac ion channel mutations leading to accelerated ventricular repolarization, arrhythmias and sudden cardiac death. Results from experimental and simulation studies suggest that changes to refractoriness and tissue vulnerability produce a substrate favorable to re-entry. Potential electromechanical consequences of the SQTS are less well-understood. The aim of this study was to utilize electromechanically coupled human ventricle models to explore electromechanical consequences of the SQTS.

**Methods and Results:** The Rice et al. mechanical model was coupled to the ten Tusscher et al. ventricular cell model. Previously validated K^+^ channel formulations for SQT variants 1 and 3 were incorporated. Functional effects of the SQTS mutations on [Ca^2+^]_*i*_ transients, sarcomere length shortening and contractile force at the single cell level were evaluated with and without the consideration of stretch-activated channel current (*I*_sac_). Without *I*_sac_, at a stimulation frequency of 1Hz, the SQTS mutations produced dramatic reductions in the amplitude of [Ca^2+^]_*i*_ transients, sarcomere length shortening and contractile force. When *I*_sac_ was incorporated, there was a considerable attenuation of the effects of SQTS-associated action potential shortening on Ca^2+^ transients, sarcomere shortening and contractile force. Single cell models were then incorporated into 3D human ventricular tissue models. The timing of maximum deformation was delayed in the SQTS setting compared to control.

**Conclusion:** The incorporation of *I*_sac_ appears to be an important consideration in modeling functional effects of SQT 1 and 3 mutations on cardiac electro-mechanical coupling. Whilst there is little evidence of profoundly impaired cardiac contractile function in SQTS patients, our 3D simulations correlate qualitatively with reported evidence for dissociation between ventricular repolarization and the end of mechanical systole.

## Introduction

The short QT syndrome (SQTS) was first recognized as a distinct clinical entity in 2000 (Gussak et al., 2000). It is characterized by an abnormally short QT interval on the ECG with a QT_C_ interval of ~320 ms or less, tall and peaked T-waves, and increased *T*_peak_ − *T*_end_ width (Anttonen et al., 2009; Patel and Pavri, 2009; Couderc and Lopes, 2010; Cross et al., 2011; Gollob et al., 2011). Patients usually have structurally normal hearts and affected families tend to exhibit histories of syncope, abbreviated atrial and ventricular refractory periods, as well as increased susceptibility to atrial and ventricular arrhythmias and sudden death (Gaita et al., 2003; Schimpf et al., 2005; Giustetto et al., 2006; Hancox et al., 2011).

There are currently six identified forms of the genetic SQTS (SQT1–SQT6). SQT1-3 result from *gain-of-function* mutations to K^+^ channel subunits. For SQT1, these mutations are to the *KCNH2* (*hERG*) gene encoding the α-subunit of the rapidly-activating delayed rectifier K^+^ channel *I*_Kr_ (Brugada et al., 2004; Hong et al., 2005a; Sun et al., 2011). The SQT2 variant arises from mutations to the *KCNQ1* gene encoding the α-subunit of the slowly-activating delayed rectifier K^+^ channel *I*_Ks_ (Bellocq et al., 2004; Hong et al., 2005b), whilst SQT3 involves mutations to the *KCNJ2* gene encoding the Kir 2.1 protein, which underlie the inwardly-rectifying K^+^ current *I*_K1_ (Priori et al., 2005; Hattori et al., 2011; Deo et al., 2013). SQT4–SQT6 are due, respectively, to *loss-of-function* mutations to the *CACNA1C, CACNB2b* (Antzelevitch et al., 2007) and *CACNA2D1* (Templin et al., 2011) genes encoding the α1C, β2b, and α2δ-1- subunits of the L-type Ca^2+^ channel.

Pro-arrhythmic mechanisms in the SQTS have been investigated through the application of K^+^ channel openers to left ventricular wedge preparations (e.g., Extramiana and Antzelevitch, 2004; Patel and Antzelevitch, 2008). Data from these experiments have been suggestive of a role for amplified transmural dispersion of repolarization and abbreviation of effective refractory period in the arrhythmogenic substrate in the SQTS (e.g., Extramiana and Antzelevitch, 2004; Patel and Antzelevitch, 2008). However, at present there are no phenotypically accurate animal models of the SQTS, making *in silico* approaches attractive for exploring the consequences of identified SQTS mutations. Computer models have reproduced QT interval shortening produced by K^+^ channel mutations in the syndrome (Zhang and Hancox, 2004; Priori et al., 2005; Weiss et al., 2005; Zhang et al., 2008; Adeniran et al., 2011, 2012; Deo et al., 2013). Using a Markov-model of the N588K-hERG SQT1 mutation based on experimental data from recombinant wild-type and N588K-hERG channels, we have recently shown that this SQT1 mutation reduces substrate size and increases tissue vulnerability to premature stimuli in order to facilitate and maintain re-entrant excitation waves in 2D and 3D tissue. We have also shown that the SQT3 D172N Kir2.1 mutation increases tissue vulnerability, alters excitability, stabilizes and accelerates re-entry (Adeniran et al., 2012).

Although the SQTS is an electrical disorder, the heart is both an electrical and mechanical organ and it is feasible, at least in principle, that abbreviated repolarization in the syndrome might influence the mechanical function of the heart. In SQTS patients, there is some evidence of significant dissociation between ventricular repolarization and the end of mechanical systole (Schimpf et al., 2008). All modeling studies to-date that have investigated arrhythmogenesis in the SQTS have utilized ventricular cell and tissue electrical models that do not consider mechanical properties (Zhang and Hancox, 2004; Priori et al., 2005; Weiss et al., 2005; Zhang et al., 2008; Adeniran et al., 2011, 2012; Deo et al., 2013). Through mechano-electric feedback, the heart is able to regulate its electrical activity in response to changes in contractility or volume load (Lab, 1982, 1996; Franz, 1996). This regulation is believed to occur through the activation of stretch-activated channels (SACs) (Taggart, 1996; Bett and Sachs, 1997; Hu and Sachs, 1997; Youm et al., 2005). As potential electromechanical consequences of the SQTS are incompletely understood, the present study was conducted in order: (1) to investigate the potential functional consequences of the SQTS on ventricular contraction at the single cell, tissue and organ levels in the presence and absence of a stretch-activated current (*I*_sac_) and (2) to evaluate the relationship between ventricular repolarization and mechanical systole in the setting of the SQTS. In order to address these aims, established models of the SQT1 and SQT3 K^+^-channel-linked SQTS variants (Adeniran et al., 2011, 2012) were coupled to a validated mechanical model (Rice et al., 2008).

## Materials and methods

### SQT1 (*I*_Kr_) and SQT3 (*I*_K1_) formulations

For SQT1, we used a biophysically-detailed Markov chain model formulation which incorporates the experimentally observed kinetic properties of wild-type (WT) and N588K-mutated hERG/*I*_Kr_ channel current at 37°C (Adeniran et al., 2011). For SQT3, we employed a biophysically-detailed Hodgkin-Huxley model formulation (Adeniran et al., 2012), which also incorporates the experimentally observed kinetic properties of the D172N-mutant Kir 2.1 channel at 37°C.

### Electromechanical model

For electrophysiology, we utilized the ten Tusscher and Panfilov (TP) human ventricular single cell model (Ten Tusscher and Panfilov, 2006), which recapitulates human ventricular cell electrical and membrane channel properties and the transmural heterogeneity of ventricular action potential (AP) across the ventricular wall (Ten Tusscher et al., 2004; Ten Tusscher and Panfilov, 2006). The TP model was modified and updated in 2006 to incorporate newly available experimental data (Xia et al., 2006); these modifications were also employed in the present study. This approach mirrors that used in our recent studies of electrical consequences of the SQT1 and SQT3 mutations (Adeniran et al., 2011, 2012).

We used the Rice et al. myocyte contraction model (Rice et al., 2008) to describe the mechanics of a cardiac myocyte. This model was chosen as it is based on the cross-bridge cycling model of cardiac muscle contraction and is able to replicate a wide range of experimental data including steady-state force-sarcomere length (F-SL), force-calcium and sarcomere length-calcium relations (Rice et al., 2008).

The intracellular calcium concentration [Ca^2+^]_*i*_ from the electrophysiology model (EP) was used as the coupling link to the myofilament mechanics model (MM). [Ca^2+^]_*i*_ produced as dynamic output from the EP model during the time course of the AP served as input to the MM model from which the amount bound to troponin is calculated. The formulation of the myoplasmic Ca^2+^ concentration in the EP model is:

(1) $$\begin{array}{l} \frac{dCa_{i}}{dt}=Ca_{\text{ibufc}}\left(\frac{V_{\text{sr}}}{V_{c}}\left(I_{\text{leak}}-I_{\text{up}}\right)+I_{\text{xfer}}\right)\\ \;-\;C_{m}\frac{I_{\text{bca}}+I_{\text{pca}}-2I_{\text{NaCa}}}{2V_{c}F} \end{array}$$

where *Ca*_ibufc_ is the total cytoplasmic buffer concentration, *V*_sr_ is the sarcoplasmic reticulum (SR) volume, *V*_*c*_ is the cytoplasmic volume, *I*_leak_ is the SR Ca^2+^ leak current, *I*_up_ is the SR Ca^2+^ pump current, *I*_xfer_ is the diffusive Ca^2+^ current current between dyadic Ca^2+^ subspace and bulk cytoplasm, *C*_*m*_ is the membrane cell capacitance per unit surface area, *I*_bCa_ is the background Ca^2+^ current, *I*_pCa_ is the plateau Ca^2+^ current, *I*_NaCa_ is the Na^+^/Ca^2+^ exchanger and *F* is the Faraday constant.

The flux of the binding of Ca^2+^ to troponin was incorporated into Equation 1 as follows:

(2) $$\begin{array}{l} \text{​​​}\frac{dCa_{i}}{dt}=Ca_{\text{ibufc}}\left(\frac{V_{\text{sr}}}{V_{c}}\left(I_{\text{leak}}-I_{\text{up}}\right)+I_{\text{xfer}}\right)\\ \;-\;C_{m}\frac{I_{\text{bCa}}+I_{\text{pCa}}-2I_{\text{NaCa}}}{2V_{c}F}-\frac{dTropTot_{\text{Ca}}}{dt}\times \frac{1}{1000} \end{array}$$

where $\frac{dTropTot_{\text{Ca}}}{dt}$ is the rate of Ca^2+^ binding to troponin. The combination of all state variables from the EP model with the MM model and the substitution of (Equation 2) for (Equation 1) yielded a human ventricular myocyte electromechanical cell model.

### Stretch-activated current

In accord with previous studies (Kohl and Sachs, 2001; Panfilov et al., 2005; Youm et al., 2005; Kuijpers, 2008; Lunze et al., 2010), we incorporated a stretch-activated current (*I*_sac_) into the electromechanics model using the following formulation:

(3) $$I_{\text{sac}}=G_{\text{sac}}\times P_{m}\times (V_{m}-E_{\text{sac}})$$

where *G*_sac_ and *E*_sac_ are the maximum channel conductance and reversal potential of the SAC, respectively. In the electromechanics model, *E*_sac_ was typically set to 1 mV and describes the experimentally observed depolarizing effect of the channel (Kohl et al., 1999; Trayanova et al., 2004). *V*_*m*_ is the membrane potential and *P*_*m*_ is the channel's open probability modeled as:

(4) $$P_{m}=\frac{1.0}{1+e^{-\left(\frac{\varepsilon -\varepsilon_{1/2}}{k_{\text{e}}}\right)}}$$

where ε and ε_1/2_ are the strain (with an explicit dependence on the sarcomere length) and half-activation strain, respectively, *K*_*e*_ = 0.02 (Zabel et al., 1996; Youm et al., 2005; Lunze et al., 2010) is the activation slope.

The SAC is assumed to be permeable to Na^+^, K^+^ and Ca^2+^ (Kamkin et al., 2000; Youm et al., 2005; Kuijpers, 2008) with *I*_sac_ therefore defined as:

(5) $$I_{\text{sac}}=I_{\text{sac},\text{ Na}}+I_{\text{sac},\text{ K}}+I_{\text{sac},\text{ Ca}}$$

where *I*_sac, Na_, *I*_sac, K_, and *I*_sac, Ca_ are the contributions of Na^+^, K^+^ and Ca^2+^ to *I*_sac_. To evaluate the effects of the permeability of the SAC to Na^+^, K^+^, and Ca^2+^, two permeability ratio cases were considered in the single cell simulations: *P*_Na_ : *P*_K_ : *P*_Ca_ = 1:1:0 and *P*_Na_ : *P*_K_ : *P*_Ca_ = 1:1:1 where *P*_Na_, *P*_K_, and *P*_Ca_ are the relative permeabilities of the channel to Na^+^, K^+^ and Ca^2+^, respectively.

### Tissue mechanics model

We modeled cardiac tissue mechanics within the theoretical framework of non-linear elasticity (Marsden and Hughes, 1994; Holzapfel, 2000) as an inhomogeneous, anisotropic, nearly incompressible non-linear material similar to previous studies (Hunter et al., 1997; Costa et al., 2001; Whiteley et al., 2007; Niederer and Smith, 2008; Pathmanathan and Whiteley, 2009). We used a two-field variational principle with the deformation *u* and the hydrostatic pressure *p* as the two fields (Lions and Ciarlet, 1994; Holzapfel, 2000; Bonet and Wood, 2008). *p* is utilized as the Lagrange multiplier to enforce the near incompressibility constraint. Thus, the total potential energy functional Π for the mechanics problem is formulated as:

(6) $$\Pi (u,p)=\Pi_{\text{int}}(u,p)+\Pi_{\text{ext}}(u)$$

where Π_int_ (*u, p*) is the internal potential energy or total strain energy of the body and Π_ext_ (*u*) is the external potential energy or potential energy of the external loading of the body. With the axes of the geometry aligned to the underlying tissue microstructure (Seemann et al., 2006; Legrice et al., 1997), the second Piola-Kirchhoff stress tensor *S*, obtained from the directional derivative of Equation 6 in the direction of an arbitrary virtual displacement and which relates a stress to a strain measure (Holzapfel, 2000; Bonet and Wood, 2008) is defined as:

(7) $$S=\frac{1}{2}\left(\frac{\partial W}{\partial E_{\text{MN}}}+\frac{\partial W}{\partial E_{\text{NM}}}\right)-pC_{\text{MN}}^{-1}+S_{\text{ActiveTension}}$$

where *W* is a strain energy function that defines the constitutive behavior of the material, *E* is the Green-Lagrange strain tensor that quantifies the length changes in a material fiber and angles between fiber pairs in a deformed solid, *C* is the Right-Cauchy green strain tensor, *p* is a Lagrange multiplier (referred to as the hydrostatic pressure in the literature) used to enforce incompressibility of the cardiac tissue, *S*_ActiveTension_ is a stress tensor incorporating active tension from the electromechanics cell model and enables the reproduction of the three physiological movements of the ventricular wall: longitudinal shortening, wall thickening and rotational twisting (MacGowan et al., 1997; Lorenz et al., 2000; Tseng et al., 2000; Bogaert and Rademakers, 2001; Cheng et al., 2008; Coppola and Omens, 2008; Lilli et al., 2013).

For the strain energy function *W*, we used the Guccione constitutive law (Guccione et al., 1991) given by:

(8) $$W=C_{1}e^{Q}$$

Where

(9) $$Q=C_{2}E_{11}^{2}+C_{3}\text{​}\left(E_{22}^{2}+E_{33}^{2}+2E_{23}^{2}\right)+2C_{4}\text{​}\left(E_{12}E_{21}+E_{13}E_{31}\right)$$

following previous work (Land et al., 2012), *C*_1_ = 0.831 kPa, *C*_2_ = 14.31, *C*_3_ = 4.49, *C*_4_ = 10. *E*_ij_ are the components of the Green-Lagrange strain tensor.

### Tissue electrophysiology model

The monodomain representation (Colli Franzone et al., 2005; Potse et al., 2006; Keener and Sneyd, 2008) of cardiac tissue was used for the electrophysiology model with a modification (the incorporation of the Right Cauchy Green deformation tensor ***C***), which allows the monodomain equation to take into account the effect of the deforming tissue, similar to previous studies (Nash and Panfilov, 2004; Whiteley et al., 2007; Pathmanathan and Whiteley, 2009):

(10) $$C_{m}\frac{dV}{dt}=-(I_{\text{ion}}+I_{\text{stim}})+\nabla \times (DC^{-1}\nabla V)$$

where *C*_*m*_ is the cell capacitance per unit surface area, *V* is the membrane potential, *I*_ion_ is the sum of all transmembrane ionic currents from the electromechanics single cell model, *I*_stim_ is an externally applied stimulus and *D* is the diffusion tensor. In simulations, intracellular conductivities in the fiber, cross-fiber and sheet directions were set to 3.0, 1.0, and 0.31525 ms mm^−1^, respectively. These gave a conduction velocity of 65 cm s^−1^ in the fiber direction along multiple cells, which is close to the value 70 cm s^−1^ observed in the fiber direction in human myocardium (Taggart et al., 2000).

### Computational methods

#### Geometry and meshes

The 3D simulations were carried out on a DT-MRI reconstructed anatomical human ventricle geometry, incorporating anisotropic fiber orientation, from a healthy 34-year-old male. This had a spatial resolution of 0.2 mm and approximately 24.2 million nodes in total and was segmented into distinct ENDO (25%), MCELL (35%), and EPI (40%) regions. The chosen cell proportion in each region is similar to those used in other studies (Gima and Rudy, 2002; Zhang et al., 2008; Adeniran et al., 2011, 2012). The conditional activation sites were determined empirically across the ventricle wall and were validated by reproducing the activation sequence and QRS complex in the measured 64-channel ECG (Keller et al., 2009) of that person.

#### Solving the electromechanics problem

The electromechanics problem consists of two sub-problems: the electrophysiology problem and the mechanics problem. The electrophysiology problem (Equation 10) was solved with a Strang splitting method (Sundnes et al., 2005) ensuring that the solution is second-order accurate. It was discretized in time using the Crank-Nicholson method (Burnett, 1987), which is also second-order accurate and discretized in space with Finite Elements (Burnett, 1987; Braess, 2007; Brenner and Scott, 2010; Ern and Guermond, 2010). *I*_ion_ in (Equation 10) represents the single cell electromechanics model from which the active tension input to the Tissue mechanics model for contraction is obtained. The system of ordinary differential equations (ODE) composing *I*_ion_ was solved with a combination of the Rush-Larsen scheme (Rush and Larsen, 1978) and the CVODE solver (Cohen and AlanHindmarsh, 1996; Hindmarsh et al., 2005).

The mechanics problem (Equation 6) was also solved using the Finite element Method using the automated scientific computing library, FEniCS (Logg et al., 2012). The resulting non-linear system of equations was solved iteratively using the Newton method to determine the equilibrium configuration of the system. The value of the Right Cauchy Green Tensor *C* was then used to update the diffusion coefficient tensor in Equation 10. Over a typical finite element domain, *P*_2_ elements (Braess, 2007; Brenner and Scott, 2010; Ern and Guermond, 2010) were used to discretize the displacement variable *u*, while the pressure variable *p* was discretized with *P*_1_ elements (Braess, 2007; Brenner and Scott, 2010; Ern and Guermond, 2010). This *P*_2_–*P*_1_ mixed finite element has been proven to ensure stability (Chamberland et al., 2010; Haga et al., 2012; Logg et al., 2012) and an optimal convergence rate (Hughes, 2000; Chamberland et al., 2010; Ern and Guermond, 2010).

The algorithm for solving the full electromechanics problem is as follows:

1. Determine the initial deformation and obtain the value of the Right Cauchy Green Tensor *C*.
2. While time < *t*_end_:
  - Solve the electrophysiology problem for Δ*t*_mechanics_ = 1 ms with *C* as input and active tension *T*_a_ as output (Δ*t*_electrophysiology_ = 0.01 ms).
  - Project *T*_a_ from the electrophysiology mesh onto the mechanics mesh.
  - Solve the mechanics problem with *T*_a_ as input and *C* as output.

## Results

### Single cell electromechanical simulations

#### Simulations without incorporation of *I*_sac_

**Figure 1:**
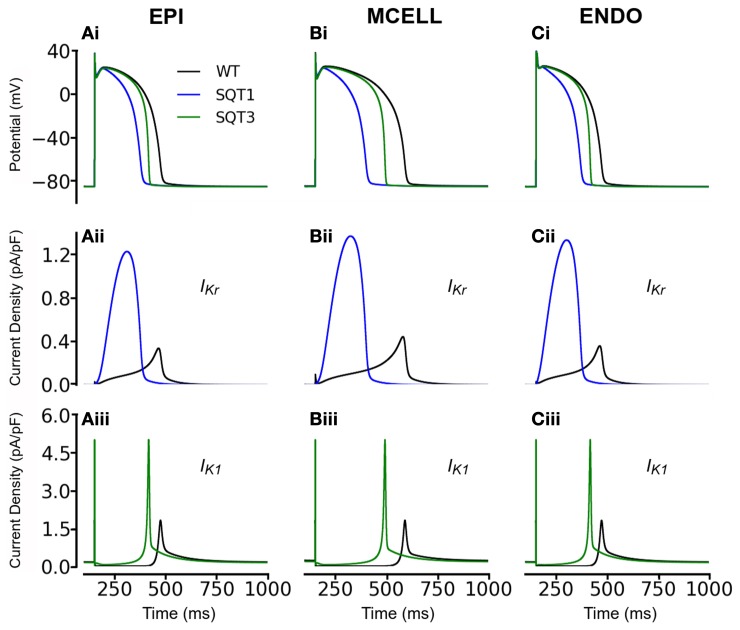
**Simulation of ventricular action potentials and the time course of *I*_Kr_ and *I*_K1_. (Ai–Ci)** Steady state (at a stimulation frequency of 1 Hz) action potentials for EPI **(Ai)**, MCELL **(Bi)**, and ENDO **(Ci)** cells under wild-type (WT; black), SQT1 (blue) and SQT3 (green) conditions. **(Aii–Cii)** Corresponding *I*_Kr_ current profiles for EPI **(Aii)**, MCELL **(Bii)**, and ENDO **(Cii)** cells under the WT (black) and SQT1 (blue) conditions. **(Aiii–Ciii)** Corresponding *I*_K1_ current profiles for EPI **(Aiii)**, MCELL **(Biii)**, and ENDO **(Ciii)** cells under the WT (black) and SQT1 (blue) conditions.

Initial simulations, in the absence of *I*_sac_, were performed using the coupled electromechanics model for the WT condition for each of ENDO, MCELL, and EPI conditions. Figure 1 shows the electrophysiological consequences of the SQT1 and SQT3 mutations in EPI, MCELL, and ENDO cell types at a stimulation frequency of 1 Hz (Figures 1Ai–Ci). For the EPI cell, action potential duration at 90% repolarization (APD_90_) was 317 ms under WT conditions and was shortened to 212 ms and 283 ms respectively under SQT1 and SQT3 conditions. For the MCELL, WT APD_90_ was 441 ms, whilst it was 232 and 382 ms under SQT1 and SQT3 conditions, respectively. For the ENDO cell model, WT APD_90_ was 317 ms, whilst it was 211 and 284 ms under SQT1 and SQT3 respectively. The observed APD shortening was more extensive for SQT1 than SQT3 and this is explicable on the basis of the relative timings and roles of *I*_Kr_ and *I*_K1_ during ventricular AP repolarization. As shown in Figures 1Aii–Cii, the SQT1 N588K mutation produced a large increase in *I*_Kr_ together with a change in the current's profile that resulted in a significant augmentation of *I*_Kr_ and shift in timing of maximal current to be earlier during the AP plateau (see also Adeniran et al., 2011). The D172N mutation significantly increased *I*_K1_ magnitude (Figures 1 Aiii–Ciii), but as *I*_K1_ contributes to terminal AP repolarization, the consequence of the mutation for APD shortening was less extensive than that for the SQT1 mutation. The electrophysiological consequences of the SQT1 and SQT3 mutations in these simulations are comparable to those reported previously from non-mechanically coupled ventricular cell models (Adeniran et al., 2011, 2012).

**Figure 2:**
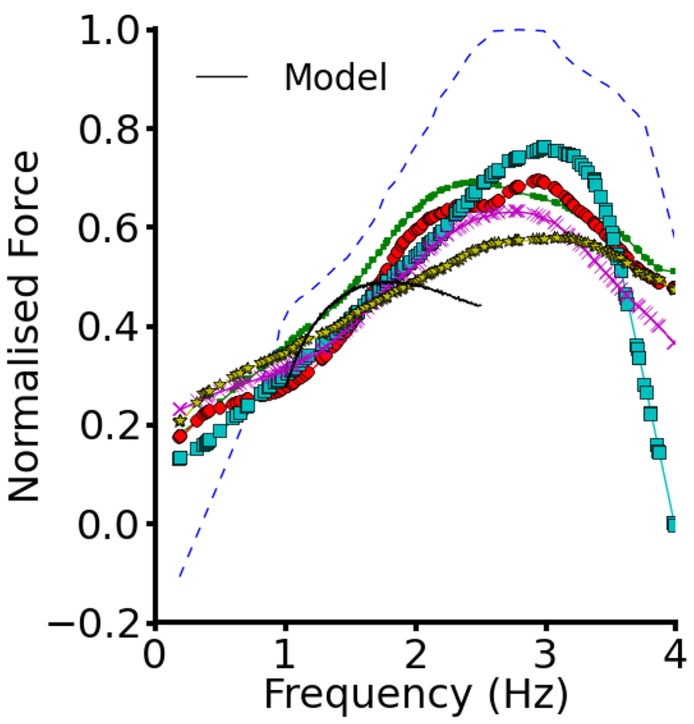
**Force-frequency relationship**. Plot of steady state normalized active force vs. heart rate using the EPI cell model. Black continuous line represents the WT electromechanics model while symbols represent experimental data from non-failing control preprarations of human myocardium. Experimental data from Mulieri et al. (1992).

**Figure 3:**
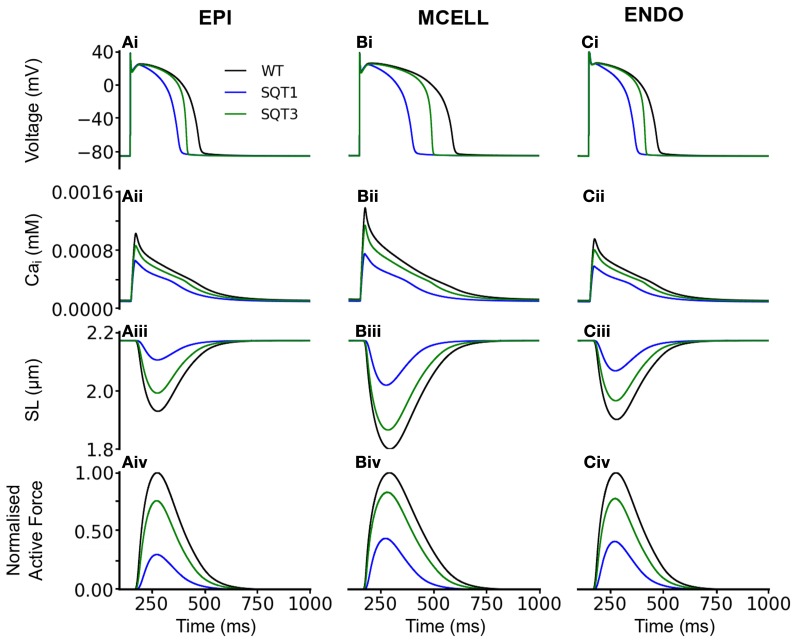
**Single cell effects of the SQT1 and SQT3 mutations (without *I*_sac_). (Ai–Ci)** WT (black), SQT1 (blue) and SQT3 (green) action potentials in the EPI **(Ai)**, MCELL **(Bi)**, and ENDO **(Ci)** cell models. **(Aii–Cii)** WT (black), SQT1 (blue) and SQT3 (green) intracellular calcium concentration and Ca^2+^ transients in the EPI **(Aii)**, MCELL **(Bii)**, and ENDO **(Cii)** cell models. **(Aiii–Ciii)** WT (black), SQT1 (blue) and SQT3 (green) sarcomere length (SL) in the EPI **(Aiii)**, MCELL **(Biii)**, and ENDO **(Ciii)** cell models. **(Aiv–Civ)** WT (black), SQT1 (blue) and SQT3 (green) active force in the EPI **(Aiv)**, MCELL **(Biv)**, and ENDO **(Civ)** cell models. Values are normalized to WT maximum active force for each cell type.

To validate the electromechanics model, we simulated force-frequency relationship (FFR) by stimulating the single cell at different frequencies for 1000 beats until steady state, recorded the maximum force developed and plotted it against frequency and compared it to experimental data (Mulieri et al., 1992). The results are shown in Figure 2. In the frequency range, 1–2 Hz, the electromechanics model produced an FFR which is qualitatively comparable to experimental data (vertical dashed lines) (Mulieri et al., 1992) and showed the Bowditch staircase or Treppe effect (Woodworth, 1902; Mulieri et al., 1992; Lakatta, 2004). All subsequent simulations in this study were carried out at 1 Hz. We then proceeded to characterize the calcium and contractile properties of the electromechanically coupled WT cell models. Figure 3 shows the action potential (AP), [Ca^2+^]_*i*_ transient, sarcomere length shortening (SLs): EPI (Figures 3Ai–Aiv), MCELL (Figures 3Bi–Biv), and ENDO (Figures 3Ci–Civ). The larger [Ca^2+^]_*i*_ transient (and hence contraction) of the MCELL compared to EPI and ENDO cells is consistent with experimental data (McIntosh et al., 2000).

Figure 3 also shows the effects of incorporating the SQT1 and SQT3 mutations on the AP, [Ca^2+^]_*i*_ transient, SL shortening and active force in the coupled electromechanics single cell models. Both mutations shortened the AP (Figures 3Ai–Ci), reduced the amplitude of [Ca^2+^]_*i*_ (Figures 3Aii–Cii) and SL shortening (Figures 3Aiii–Ciii) in each of the EPI, MCELL, and ENDO cell models. These effects led to the attenuation of contractility (percentage of WT) in all the cell types (Figures 3Aiv–Civ) (SQT1 EPI 30%; SQT3 EPI 76%; SQT1 MCELL 44%; SQT3 MCELL 83%; SQT1 ENDO 41%; SQT3 ENDO 78%). As identified in Figure 1, the effects for the SQT3 mutation were not as pronounced as the SQT1 mutation because of the relative timing of *I*_Kr_ and *I*_K1_ during the AP, with the SQT3 mutation influencing only terminal repolarization and consequently giving rise to longer APDs across the ventricular wall.

**Figure 4:**
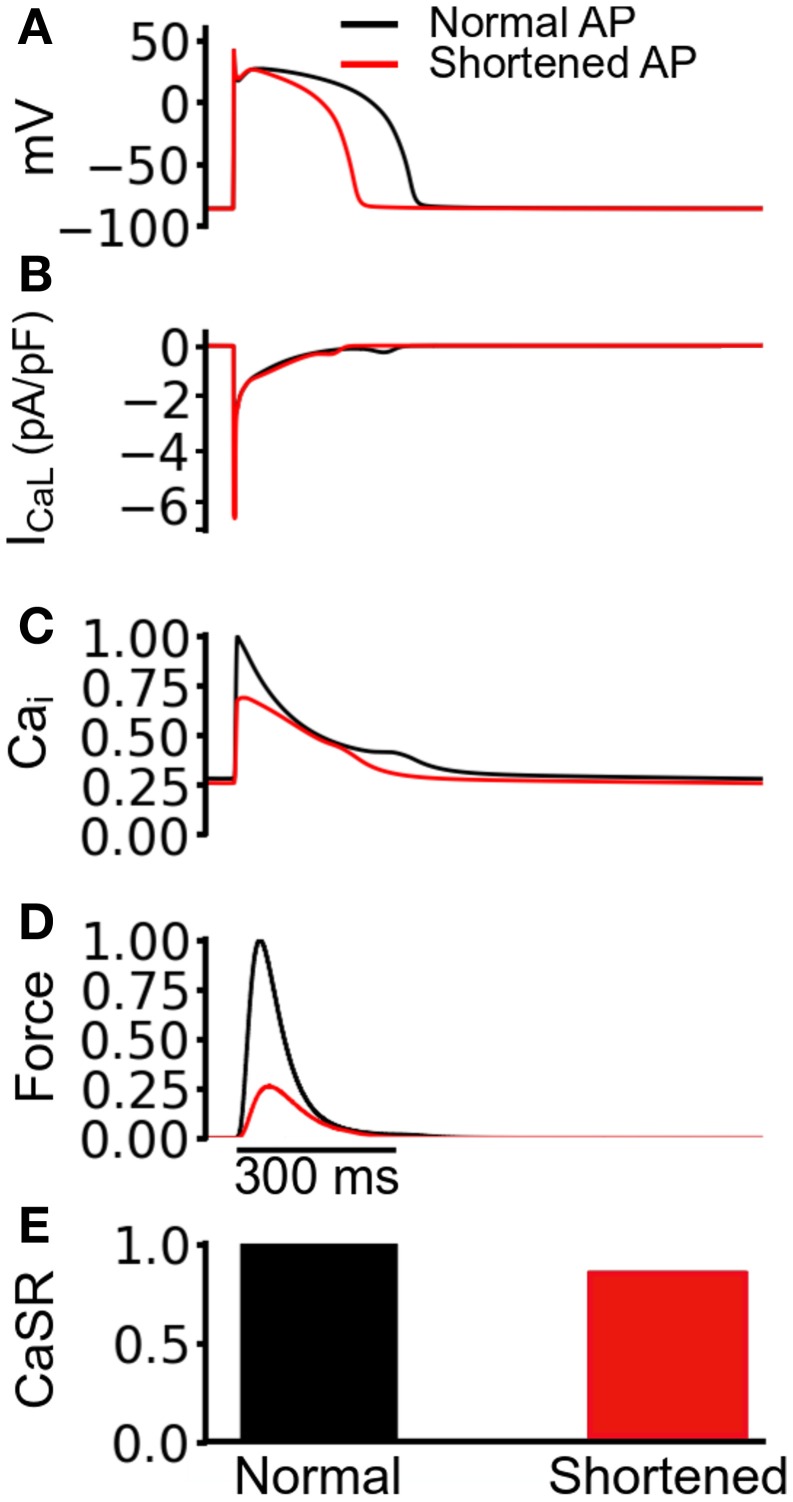
**Simulated AP clamp using the WT electromechanics model without *I*_sac_. (A)** The normal (black) and shortened (red) AP waveforms applied as voltage clamp commands to the WT electromechanics model. **(B)** *I*_CaL_ elicited by the two AP waveforms in **(A)**. **(C)** Normalized [Ca^2+^]_*i*_ elicited by the two AP waveforms in **(A)**. Values are normalized to maximum [Ca^2+^]_*i*_ for the “normal” AP waveform. **(D)** Normalized SL shortening elicited by the two AP waveforms in **(A)**. Values are normalized to maximum SL shortening for the normal AP waveform. **(E)** Normalized contractile force elicited by the two AP waveforms in **(A)**. Values are normalized to maximum active force for the normal AP waveform.

The observed reduction in the active force under the mutation conditions in Figure 3 was profound, particularly in the case of SQT1. In order to elucidate the mechanism causing such a decrease in contractile force, we performed a simulated “AP clamp” experiment on the WT electromechanics model (Figure 4), using two different AP profiles—one AP of a normal duration and the other AP with an abbreviated duration. In this experiment, WT *I*_Kr_ and *I*_K1_ formulations were used, therefore any observed alterations to[Ca^2+^]_*i*_ and contractile force would relate to APD *per se*. Figure 4A shows the two AP clamp commands used. Figure 4B shows the AP-evoked *I*_CaL_ whilst Figures 4C–E respectively show [Ca^2+^]_*i*_, active force, and the difference in steady state level of free calcium concentration in the sarcoplasmic reticulum (CaSR). The results of these simulations showed that though the shorter AP did not alter notably the peak amplitude of *I*_CaL_, it reduced the amplitude of the [Ca^2+^]_*i*_ transient, SL shortening and the active force and—notably—SR calcium content (CaSR). These effects are similar to the results shown in Figure 3 for the SQT models. However, in the AP clamp simulation, any observed reduction in amplitude of [Ca^2+^]_*i*_, SL shortening, CaSR and the active force can be attributed solely to consequences of application of the shorter AP waveform. This suggests that the key reason for the reduced active force in the SQTS setting (Figure 3) is the indirect effect of the SQT mutation-linked AP shortening on Ca^2+^ handling (and on SR content in particular).

**Figure 5:**
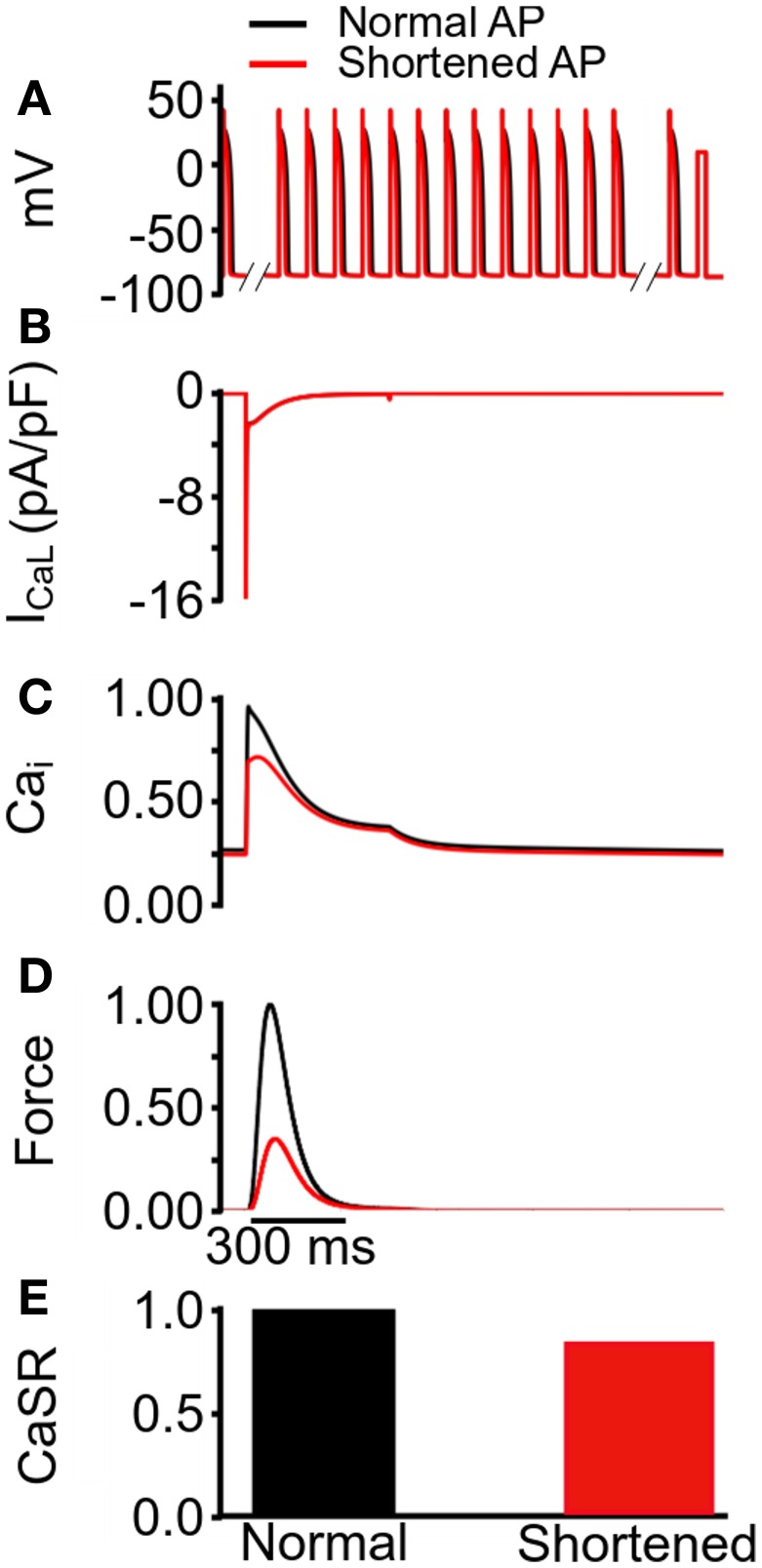
**Changes in steady state SR content induced by conditioning trains of action potentials of differing duration. (A)** Protocol used to determine steady state SR content, comprised of train of 100 normal (black) and shortened (red) AP clamp commands followed by a 300 ms square command voltage pulse to +10 mV. **(B)** *I*_CaL_ elicited by the +10 mV voltage command. Peak *I*_CaL_ is equal with the two AP waveforms in **(A)**. **(C)** Normalized [Ca^2+^]_*i*_ elicited by the by the +10 mV voltage command. Values are normalized to maximum [Ca^2+^]_*i*_ for the “normal” AP waveform. **(D)** Normalized active force elicited by the +10 mV voltage command. Values are normalized to maximum active force for the normal AP waveform. **(E)** Normalized maximum SR Ca^2+^ content prior to the application of +10 mV voltage command in **(A)**. Values are normalized to maximum SR Ca^2+^ for the normal AP waveform.

To investigate further the functional impact of AP duration on the loading of SR calcium content at the steady state, we applied conditioning trains containing one of two different AP clamp commands (one with a longer and the other with a shorter AP duration) to the WT electromechanics model; the conditioning train was followed by an identical single square voltage command to +10 mV for 300 ms (Figure 5A). With the conditioning train of APs comprised of the longer duration AP, it was observed that the +10 mV square pulse command produced a larger [Ca^2+^]_*i*_ transient than that when conditioning trains of shorter duration APs were applied. Results are shown in Figure 5C. With the conditioning AP trains of different durations, the square pulse elicited an identical *I*_CaL_ (Figure 5B), but a smaller [Ca^2+^]_*i*_ amplitude (Figure 5C) and active force (Figure 5D) for the shorter duration AP. These simulations also showed that prior to the square pulse command, the SR was filled to a greater level with the longer duration conditioning APs than with those of shorter duration,(as illustrated by the steady state CaSR in Figure 5E). This further validates the notion that the attenuation of [Ca^2+^]_*i*_ amplitude and contractility with the SQT mutations was consequent upon reduced SR content associated with abbreviation of AP duration.

#### Incorporation of *I*_sac_

**Figure 6:**
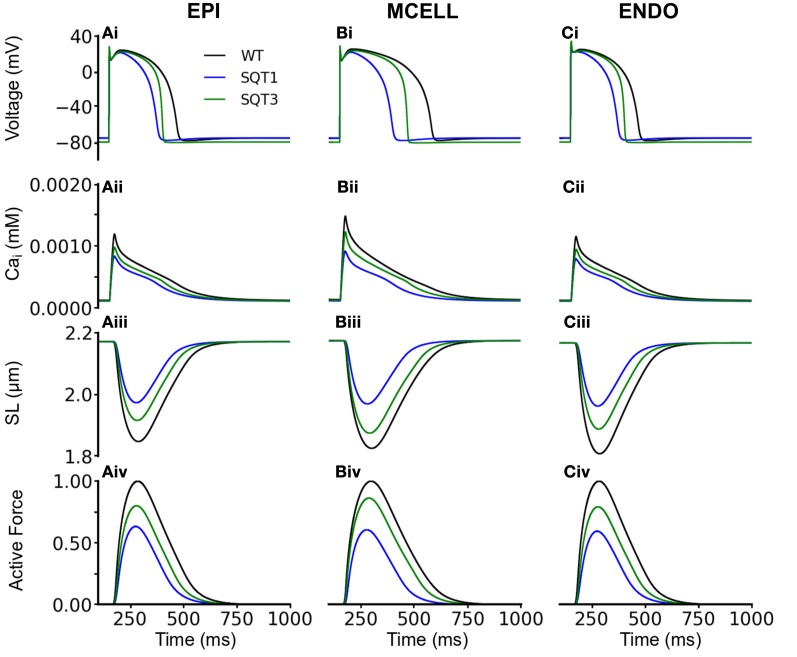
**Effects of *I*_sac_ on the WT, SQT1, and SQT3 electromechanics model. (Ai–Ci)** WT (black), SQT1 (blue) and SQT3 (green) action potentials in the EPI **(Ai)**, MCELL **(Bi)**, and ENDO **(Ci)** cell models. **(Aii–Cii)** WT (black), SQT1 (blue) and SQT3 (green) intracellular calcium concentration and Ca^2+^ transients in the EPI **(Aii)**, MCELL **(Bii)**, and ENDO **(Cii)** cell models. **(Aiii–Ciii)** WT (black), SQT1 (blue) and SQT3 (green) sarcomere length (SL) in the EPI **(Aiii)**, MCELL **(Biii)**, and ENDO **(Ciii)** cell models. **(Aiv–Civ)** WT (black), SQT1 (blue) and SQT3 (green). SAC in these simulations were permeable to Na^+^, K^+^, and Ca^2+^ in the ratio 1:1:0.

**Figure 7:**
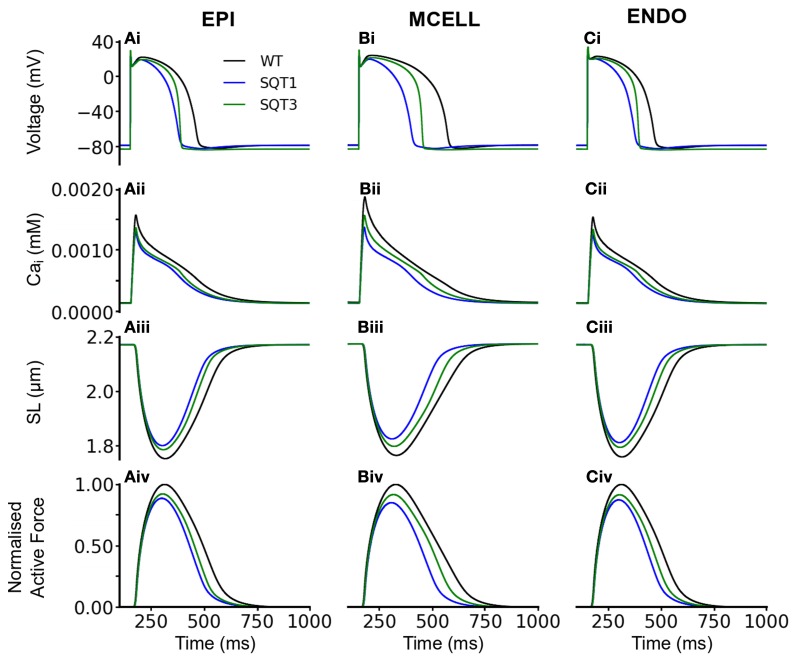
**Effects of *I*_sac_ on the WT, SQT1, and SQT3 electromechanics model. (Ai–Ci)** WT (black), SQT1 (blue) and SQT3 (green) action potentials in the EPI **(Ai)**, MCELL **(Bi)**, and ENDO **(Ci)** cell models. **(Aii–Cii)** WT (black), SQT1 (blue) and SQT3 (green) intracellular calcium concentration and Ca^2+^ transients in the EPI **(Aii)**, MCELL **(Bii)**, and ENDO **(Cii)** cell models. **(Aiii–Ciii)** WT (black), SQT1 (blue) and SQT3 (green) sarcomere length (SL) in the EPI **(Aiii)**, MCELL **(Biii)**, and ENDO **(Ciii)** cell models. **(Aiv–Civ)** WT (black), SQT1 (blue) and SQT3 (green). SAC in these simulations were permeable to Na^+^, K^+^, and Ca^2+^ in the ratio 1:1:1.

We then performed comparable simulations with the incorporation of *I*_sac_. Figures 6, 7 show the results with the SAC assumed to be permeable to Na^+^, K^+^ and Ca^2+^ in the ratio 1:1:0 (Figure 6) and 1:1:1 (Figure 7). The resting potential for EPI, MCELL and ENDO decreased from −86 to −76 mV (*I*_sac_ at 1:1:0 permeability ratio) and to −79 mV (*I*_sac_ at 1:1:1 permeability ratio) for the WT and SQT1 conditions respectively. Depolarization of the membrane potential is an effect of SACs, which has been observed experimentally (Boland and Troquet, 1980; Franz et al., 1992; Kamkin et al., 2000). The resting membrane potential remained unchanged under the SQT3 condition because the increase in outward *I*_K1_ caused by the mutation counteracted the depolarizing effect of *I*_sac_. Similar to the situation without *I*_sac_, the incorporation of SQT1 and SQT3 mutations abbreviated the AP in all three cell types (Figures 6Ai–Ci, 7Ai–Ci). The most significant consequences of inclusion of *I*_sac_ were upon [Ca^2+^]_*i*_ and contractile activity. Thus, the results shown in Figures 6, 7 indicate that incorporation of *I*_sac_ attenuated the reduction caused by the SQT1 and SQT3 mutations shown in Figure 3 on [Ca^2+^]_*i*_ (Figures 6Aii–Cii, 7Aii–Cii), SLs (Figures 6Aiii–Ciii, 7Aiii–Ciii) and active force (Figures 6Aiv–Civ, 7Aiv–Civ). *I*_sac_ incorporated at 1:1:1 permeability ratio (i.e., incorporating Ca^2+^ permeability) produced the greater effect, with contractility across the ventricular wall being approximately 85% of control under the SQT1 mutation and 92% of control under the SQT3 mutation. In contrast, with *I*_sac_ incorporated at a permeability ratio of 1:1:0, on average across the ventricular wall, the contractile force was 62% of control under the SQT1 condition and 82% of control under the SQT3 condition.

**Figure 8:**
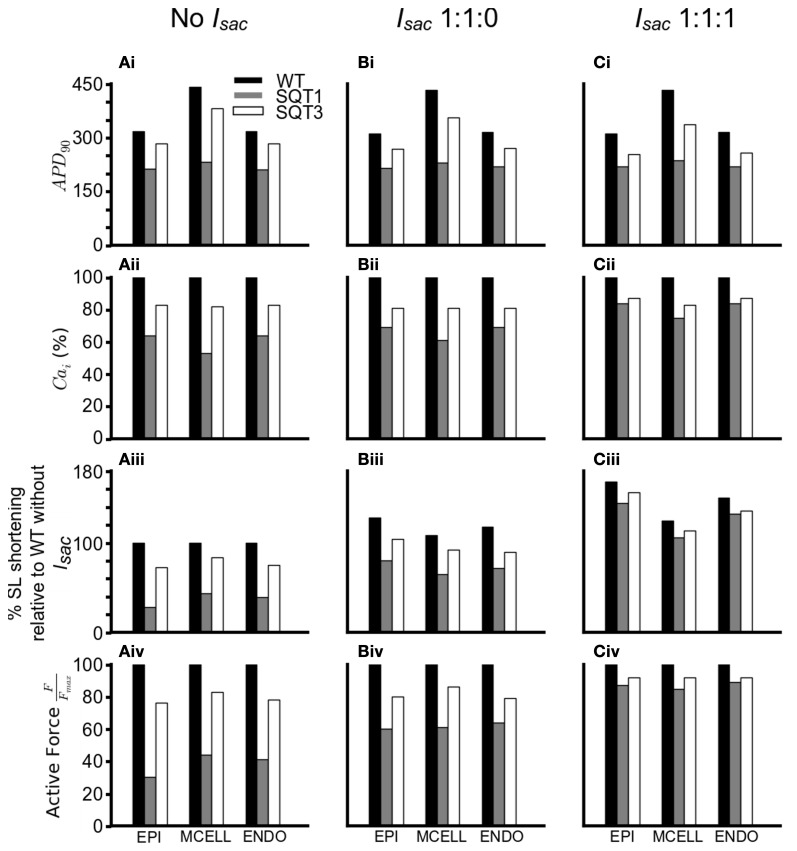
**Summary of effects of SQTS mutations on APD_90_, [Ca^2+^]_*i*_, SL shortening and active force under different simulation conditions. (Ai–Ci)** Changes in APD_90_under the WT (black), SQT1 (gray) and SQT3 (white) in the EPI, MCELL and ENDO cell types without *I*_sac_ **(Ai)**, with *I*_sac_ at a permeability ratio of 1:1:0 **(Bi)** and with *I*_sac_ at a permeability ratio of 1:1:1 **(Ci)**. **(Aii–Cii)** Percentage changes in [Ca^2+^]_*i*_ under the WT (black), SQT1 (gray) and SQT3 (white) in the EPI, MCELL and ENDO cell types without *I*_sac_ **(Aii)**, with *I*_sac_ at a permeability ratio of 1:1:0 **(Bii)** and with *I*_sac_ at a permeability ratio of 1:1:1 **(Cii)**. **(Aiii–Ciii)** Percentage sarcomere length shortening under the WT (black), SQT1 (gray) and SQT3 (white) in the EPI, MCELL, and ENDO cell types without *I*_sac_ **(Aiii)**, with *I*_sac_ at a permeability ratio of 1:1:0 **(Biii)** and with *I*_sac_ at a permeability ratio of 1:1:1 **(Ciii)**. Values are relative to WT without *I*_sac_ **(Aiii)**. **(Aiv–Civ)** Normalized active force under the WT (black), SQT1 (gray) and SQT3 (white) in the EPI, MCELL and ENDO cell types without *I*_sac_ **(Aiv)**, with *I*_sac_ at a permeability ratio of 1:1:0 **(Biv)** and with *I*_sac_ at a permeability ratio of 1:1:1 **(Civ)**.

In order to investigate how *I*_sac_ attenuated the effects of the SQT1 and SQT3 mutations on [Ca^2+^]_*i*_ and cell contractility, a side-by-side comparison was made between the effects of the SQT1 and SQT3 mutations on AP duration, [Ca^2+^]_*i*_ and force production, in the absence of *I*_sac_ and with *I*_sac_ incorporated at the two permeability ratios (Figure 8). Figures 8Ai–Ci shows that the incorporation of *I*_sac_ at both permeability ratios reduced the APDs under the WT, SQT1 or SQT3 conditions, with a greater APD reduction in the case of permeability ratio of 1:1:1 than that of 1:1:0 as shown in Table 1. There was a greater [Ca^2+^]_*i*_ transient amplitude under both SQTS mutation conditions with the incorporation of *I*_sac_; the greatest amplitude being at the 1:1:1 permeability ratio (Figures 8Aii–Cii). From Figures 8Aiii–Ciii, it is clear to see the increase in the [Ca^2+^]_*i*_ produced a greater SL shortening (relative to WT) on incorporation of *I*_sac_, which consequently led to greater cell contractility in the SQT1 and SQT3 mutations particularly with a permeability ratio of 1:1:1 (Figures 8Aiv–Civ).

**Table 1:** **Changes in APD in the EPI, MCELL, and EDO cells with *I*_sac_**.

		**APD (ms)**		
		**No *I*_sac_**	***I*_sac_ (Permeability 1:1:0)**	***I*_sac_ (Permeability 1:1:1)**
WT	EPI	317	310	306
	MCELL	441	433	420
	ENDO	317	314	310
SQT1	EPI	212	214	218
	MCELL	232	230	237
	ENDO	211	218	218
SQT3	EPI	283	269	253
	MCELL	382	355	336
	ENDO	284	270	257

**Figure 9:**
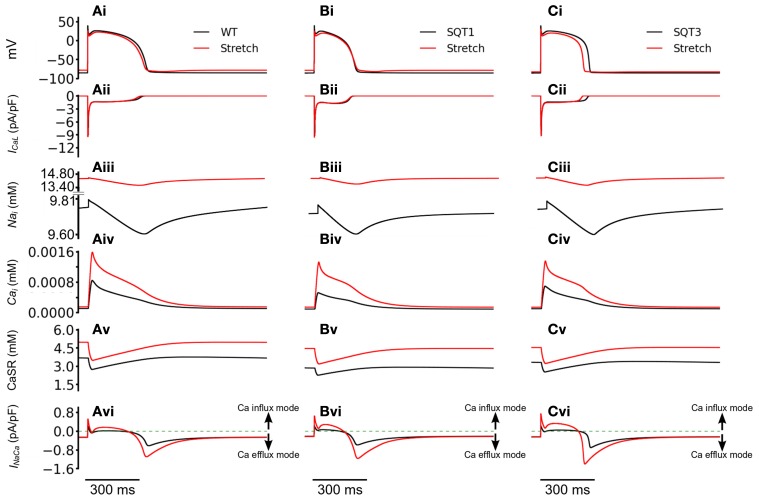
**Reverse mode operation of NCX with the incorporation of SAC. (Ai–Ci)** Action potentials of WT **(Ai)**, SQT1 **(Bi)**, and SQT3 **(Ci)** without stretch (black) and with stretch (red). **(Aii–Cii)** [Na^+^]_*i*_ for WT **(Aii)**, SQT1 **(Bii)**, and SQT3 **(Cii)** without stretch (black) and with stretch (red). **(Aiii–Ciii)** Action potentials of WT **(Aiii)**, SQT1 **(Biii)**, and SQT3 **(Ciii)** without stretch (black) and with stretch (red). As the SAC is permeable to Na^+^, it is of higher amplitude under stretch conditions. **(Aiv–Civ)** [Ca^2+^]_*i*_ for WT **(Aiv)**, SQT1 **(Biv)**, and SQT3 **(Civ)** without stretch (black) and with stretch (red). **(Av–Cv)** SR Ca^2+^ release under WT **(Av)**, SQT1 **(Bv)** and SQT3 **(Cv)** without stretch (black) and with stretch (red). The SR is refilled to a greater level prior to AP initiation under stretch conditions. **(Avi–Cvi)** *I*_NaCa_ of WT **(Avi)**, SQT1 **(Bvi)** and SQT3 **(Cvi)** without stretch (black) and with stretch (red).

We then investigated how the incorporation of *I*_sac_ led to better maintenance of the [Ca^2+^]_*i*_ transient magnitude. Figure 9 shows the computed APs (Figures 9Ai–Ci), *I*_CaL_ (Figures 9Aii–Cii), [Na^+^]_*i*_ (Figures 9Aiii–Ciii), [Ca^2+^]_*i*_ (Figures 9Aiv–Civ), CaSR (Figures 9 Av–Cv) and *I*_NaCa_ (Figures 9 Avi–Cvi) with and without *I*_sac_ (permeability ratio 1:1:1) in the WT, SQT1 and SQT2 conditions. Under the WT, SQT1 and SQT3 conditions, it was shown that incorporation of *I*_sac_ did not produce a noticeable change in the amplitude of *I*_CaL_, but elevated [Na^+^]_*i*_, [Ca^2+^]_*i*_, and CaSR. These changes in the intracellular Na^+^ and Ca^2+^ concentrations were associated with an altered *I*_NaCa_ as shown in Figures 9Avi–Cvi. In the case when *I*_sac_ was absent, during the initial depolarization phase of the AP, *I*_NaCa_ operated briefly in its reverse-mode that brought Ca^2+^ into the cytoplasmic space, producing an outward *I*_NaCa_. During the plateau and early repolarization phases, *I*_NaCa_ remained almost zero for a period before switching to a forward mode to extrude Ca^2+^ out of cell cytoplasmic space, producing an inward *I*_NaCa_ current in the late repolarization phase. However, in the case with *I*_sac_, the activation of *I*_sac_ brought more Na^+^ into the cell cytoplasmic space (as it is permeable to Na^+^) producing an elevated level of [Na^+^]_*i*_ (Figures 9Aiii–Ciii) as compared to the case when *I*_sac_ was absent. Consequently, *I*_NaCa_ operated longer in a reverse-mode during the AP phase before it reverted to a normal mode in late repolarization phase. This led to a greater *I*_NaCa_ amplitude in both the reverse and forward modes (Figures 9 Avi–Cvi). A greater *I*_NaCa_ in the reverse-mode brought more Ca^2+^ into the cell cytoplasmic space, resulting in a higher systolic level of [Ca^2+^]_*i*_ (Figures 9 Aiv–Civ) and a greater level of the CaSR (Figures 9Av–Cv). Though this observation was *qualitatively* similar for the WT (Figure 9Avi), SQT1 (Figure 9Bvi) and SQT3 conditions (Figure 9Cvi), in *quantitative* terms the increase in the [Ca^2+^]_*i*_ was more dramatic in the SQT1 and 3 than WT settings. Thus, incorporation of *I*_sac_ into the simulations increased [Ca^2+^]_*i*_ by 88% under the WT condition, but by 153% under the SQT1 condition and by 94% under the SQT3 setting. The greater increase of [Ca^2+^]_*i*_ under the SQT simulation conditions provides an explanation for the maintenance of the Ca^2+^ transient by *I*_sac_. Our simulated elevation of [Na^+^]_*i*_ by *I*_sac_ is consistent with previous experimental studies (Alvarez et al., 1999; Isenberg et al., 2003; Youm et al., 2005) that have shown an increase in cytosolic and total [Na^+^]_*i*_ by a mechanical stretch in human, mouse and ventricular myocytes, which has been attributed to the reverse-mode of *I*_NaCa_ during the rising phase of APs (Gannier et al., 1996; Alvarez et al., 1999; Calaghan and White, 1999; Kamkin et al., 2000, 2003; Calaghan et al., 2003; Youm et al., 2005).

### 3D simulations

**Figure 10:**
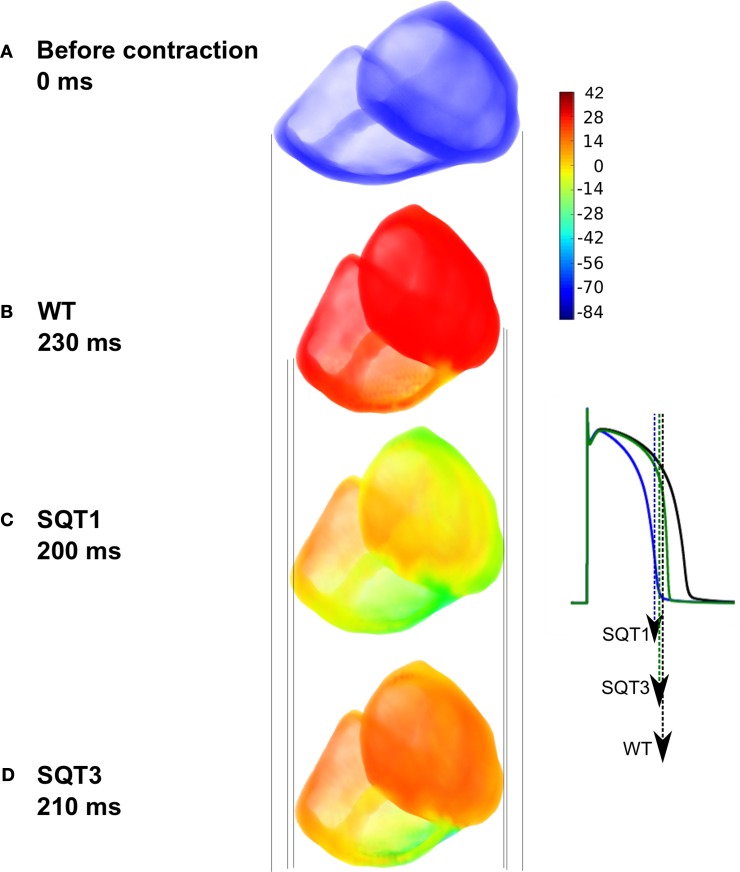
**Electromechanical coupling in 3D ventricle model under the SQT1 and SQT3 mutations with *I*_sac_. (A)** Resting position of the ventricles prior to electrical stimulation. **(B)** Snapshot of maximum deformation occurring at 230 ms under the WT condition just at the onset of repolarization (black AP). **(C)** Snapshot of maximum deformation occurring at 200 ms under the SQT1 condition. Repolarization is already significantly advanced (blue AP). **(D)** Snapshot of maximum deformation occurring at 210 ms under the SQT3 condition. Repolarization is already in progress (green AP). Vertical lines show a comparison of the degree of contraction of the ventricles between the different conditions. Color bar represents the membrane potentials of cells in the ventricles ranging from –86 to 42 mV. The APs shown in the inset are from a left ventricular cell under the WT, SQT1 and SQT3 conditions. Arrows indicate the snapshot time shown in the main figure for each condition corresponding to the repolarization time at which maximal deformation occurs.

Results from single cell models cannot be translated automatically to the intact tissue situation due to intercellular electrical coupling and mechanical deformation of tissue. Schimpf et al. (2008) observed a dissociation between ventricular repolarization and the end of mechanical systole in SQT patients. Consequently, to investigate this observation, we implemented a multi-cellular 3D tissue model of the human ventricles that considered the intercellular electrical coupling and mechanical deformation of tissue. Simulation results using the human ventricle 3D model are shown in Figure 10. Figure 10A shows the ventricles during diastole before contraction, whilst Figure 10B shows deformation under the WT condition; maximum deformation occurred at 230 ms. Maximum deformation occurred at 200 ms and 210 ms under the SQT1 (Figure 10C) and SQT3 (Figure 10D) conditions respectively but in contrast to WT, repolarization had already advanced significantly, particularly under the SQT1 condition. The vertical lines show that contraction was greatest in the WT condition (Figure 10B) and least in the SQT1 setting (Figure 10C) but due to the incorporation of *I*_sac_, contractility was not significantly impaired in either mutation condition, which agrees with available clinical evidence (Schimpf et al., 2008).

## Discussion

### Summary of major findings

Electromechanical coupling in the heart is an active area of research and an important mechanism that couples electrical and mechanical processes is the presence of cardiac ion channels activated by mechanical stimuli such as changes in cell volume or cell stretch (Morris, 1990; Bustamante et al., 1991; Hagiwara et al., 1992; Van Wagoner, 1993; Suleymanian et al., 1995). In the present study, we have developed a family of multi-physical scale models for simulating the electromechanical coupling in the human ventricle at cellular and tissue levels under both WT and SQTS mutation conditions. Using these models we investigated the functional impact of AP abbreviation due to the SQT1 and SQT3 mutations on human ventricular mechanical dynamics. In the heart, SACs transduce mechanical energy into cellular responses and can carry considerable currents (Franz et al., 1992; Alvarez et al., 1999; Calaghan and White, 1999; Calaghan et al., 2003; Youm et al., 2005). Consequently, we incorporated a stretch-activated channel current (*I*_sac_) into our single cell models to investigate the consequences of its inclusion under WT and SQTS mutation conditions. Our simulations suggest that: (i) at least *in silico*, abbreviated repolarization in the SQTS has the potential to reduce ventricular mechanical function; (ii) the inclusion of (*I*_sac_) in the model acts to maintain the normal amplitude of the contractile force (Figures 6–8); and (iii) there is a dissociation between ventricular repolarization and the end of mechanical systole in 3D SQTS simulations (Figure 10), which matches clinical observations by Schimpf et al. (2008). Several aspects of our findings merit more detailed discussion.

### Mechanistic insights

The results of simulated AP clamp experiments utilizing longer and shorter duration APs in the WT electromechanics model (Figure 4) provide mechanistic insight into the cause of the profound reduction and effects on contractility under simulated SQT1 and SQT3 conditions. In these simulations it was shown that markedly reduced contractility was attributable to reduced SR Ca^2+^ loading. AP shortening alters cellular electrical dynamics and provides less time for SR Ca^2+^ loading and therefore SR Ca^2+^ content is compromised. This leads to a reduced SR Ca^2+^ release and, consequently, cell shortening. These observations are somewhat similar to previously reported effects of K-ATP channel openers. For example, K-ATP channel activation with lemakalim has been reported to reduce ventricular myocyte Ca^2+^ transients and contraction (Jiang et al., 1994), whilst a second K-ATP channel opener HOE 234 produced a negative inotropic effect on papillary muscle preparations (Kocić and Siluta, 1995).

Our simulation data are suggestive that the presence of SACs attenuates the reduced ventricular cell contractility arising from SQTS K channel mutations. This can be ascribed to the effects of *I*_sac_ on SR Ca^2+^ loading and therefore the amplitude of [Ca^2+^]_*i*_ transients as shown in Figure 8. With *I*_sac_, with a Na:K:Ca ratio of either 1:1:0 or 1:1:1, there was a greater [Ca^2+^]_*i*_ transient amplitude and higher SR Ca^2+^ content, resulting in a greater shortening of the SL and active force as compared to the case when *I*_sac_ was absent. Such an effect of *I*_sac_ on the intracellular Ca^2+^ handling is due to two factors. First, during the depolarization phase of the AP, *I*_NaCa_ operates in a reverse mode that brings Ca^2+^ into the cytoplasmic space due to Na^+^ influx. As *I*_sac_ is permeable to Na^+^, the activation of *I*_sac_ elevates [Na^+^]_*i*_, consequentially produces a greater reversed *I*_NaCa_ that elevates the [Ca^2+^]_*i*_ (Figure 9). The elevation of [Na^+^]_*i*_ leading to the reverse-mode of *I*_NaCa_ have been reported by previous studies (Bassingthwaighte et al., 1976; Eisner et al., 1983; Hume and Uehara, 1986; Barcenas-Ruiz et al., 1987; Gannier et al., 1996; Alvarez et al., 1999; Calaghan and White, 1999; Kamkin et al., 2000, 2003; Calaghan et al., 2003; Youm et al., 2005). Secondly, the increased [Ca^2+^]_*i*_ and the Ca^2+^ entry via *I*_NaCa_ in its reversed mode can both lead to more Ca^2+^ being pumped back to the SR, contributing to a greater CaSR, and trigger more SR Ca^2+^ release (Leblanc and Hume, 1990; Levesque et al., 1991; Litwin et al., 1996; Bers, 2001), thereby elevating [Ca^2+^]_*i*_ (Figure 10).

The dissociation between ventricular repolarization and the end of mechanical systole reflects the difference time course of the two processes. Our simulation data show that relative to ongoing mechanical contraction, ventricular repolarization terminates significantly earlier in SQTS conditions. Thus, accelerated repolarization in the SQTS exacerbates differences between electrical and mechanical events. By way of illustration, in our 3D anatomical human ventricle simulations, at the point of maximum deformation, repolarization was already underway in the SQT1 and SQT3 conditions (Figure 10) whereas it had not begun under the WT condition.

### Relevance to previous studies

Our simulation data suggest that *I*_sac_ plays an important role in modulating cardiac electromechanical coupling. This is consistent with previous findings (Hirabayashi et al., 2008; Keldermann et al., 2010). In their study, Keldermann developed a coupled electromechanical model for the left human ventricle, and used the model to investigate possible functional roles of *I*_sac_ on the re-entrant electrical wave conductions. It was found that mechanoelectrical feedback via *I*_sac_ can induce the deterioration of an otherwise stable spiral wave into turbulent wave patterns similar to that of ventricular fibrillation. A similar role for *I*_sac_ has also been observed in the study of (Hirabayashi et al., 2008). Findings from the present study add to these previous studies, in demonstrating the important role of *I*_sac_ in cardiac electromechanical dynamics.

In relation to the SQTS, Gaita et al. (2003) performed echocardiography, cardiac MRI and stress tests on the two families in which the SQTS was first reported (Gaita et al., 2003) and found no evident structural abnormalities. In subsequent work on mechanical function in the SQTS by Schimpf et al. (2008), no significant difference was seen between control subjects and SQTS patients in end systolic volume, end diastolic volume and ejection fraction. However, a dissociation between ventricular repolarization and the end of mechanical systole was observed. Our 3D simulations (Figure 10) qualitatively match and substantiate this clinical finding (Schimpf et al., 2008).

### Limitations

In addition to acknowledged limitations of both the TP electrophysiology model (Ten Tusscher and Panfilov, 2006) and the Rice et al. (2008) mechanics model, although our coupled electromechanics model exhibited the Bowditch staircase or Treppe effect (Woodworth, 1902; Mulieri et al., 1992; Lakatta, 2004), it was only qualitatively able to reproduce experimental force-frequency characteristics. In simulations at increased pacing rates from 1.5 to 4 Hz, we observed APD shortening with an increase in the pacing rate, but due to reduced time for Ca^2+^ extrusion and SR accumulation between successive APs, there was an increase in the amplitude of the [Ca^2+^]_*i*_ transient and the active force in both WT and SQT settings. This was particularly the case at the faster rates examined, where there was insufficient time for restoration of Ca^2+^ dynamics between successive APs. These modeling observations require further validation and, if necessary, improvement in Ca^2+^ dynamics when experimental data become available. However, over the frequency range of 1–2 Hz our data matched reasonably experimental force-frequency data (Figure 2) and all simulations of the effects of SQT mutations presented here were conducted at 1 Hz. In the ventricular electrophysiology cell models, we did not consider the effects of β-adrenergic stimulation or more physiologically-detailed Ca^2+^ handling mechanisms as implemented in some recently published models (Grandi et al., 2010; O'Hara et al., 2011). These effects may affect quantitatively the simulation results (Puglisi et al., 2013). Additionally, due to lack of experimental data on the *I*_sac_ in human ventricular myocytes, *I*_sac_ density was based on the study of (Panfilov et al., 2005; Youm et al., 2005; Kuijpers, 2008; Kohl and Sachs, 2001; Lunze et al., 2010). Whilst we have investigated the effects of *I*_sac_ on attenuation of force reduction in the SQTS setting, it is possible that alternative mechanisms may be involved such as calcium transport controlled by feedback of SR filling via store-operated Ca^2+^ channels (SOC) (Kusters et al., 2005; Kowalewski et al., 2006; Berna-Erro et al., 2012). Consequently, in additional simulations (data not shown), we have incorporated into the model a SOC channel current based on the Kuster et al. model (Kusters et al., 2005). In contrast to our findings with *I*_sac_, with *I*_*SOC*_ incorporation no significant attenuation (<6%) of the force reduction in the SQTS settings was observed for the maximal SOC channel conductance varying from 0.2 to 20 pS/pF. Whilst it is important that these potential limitations are stated, they do not fundamentally alter the principal conclusions of this study.

## Conclusion

Our tissue simulations qualitatively reproduce and provide a possible explanation for dissociation between the end of mechanical systole and ventricular repolarization (Schimpf et al., 2008): accelerated repolarization under SQTS conditions exacerbates differences in time-course between mechanical and electrical events. The results of the simulations in this study also raise a question as to whether electromechanical coupling involving *I*_sac_ offsets a negative inotropic effect of ventricular action potential abbreviation that might otherwise occur for K^+^-channel linked SQTS. If, *in vivo, I*_sac_ does not execute such a role, then it is possible that other compensatory changes exist in SQTS patients as accelerated repolarization might otherwise result in altered SR Ca^2+^ loading and a reduction in contractile activity.

### Conflict of interest statement

The authors declare that the research was conducted in the absence of any commercial or financial relationships that could be construed as a potential conflict of interest.
